# Means and ends of effective global risk assessments for genetic pest management

**DOI:** 10.1186/s12919-018-0112-2

**Published:** 2018-07-19

**Authors:** Geoff Turner, Camilla Beech, Lucia Roda

**Affiliations:** 1 Imperial College London, Silwood Park, Ascot, UK; 2 Formerly Oxitec Ltd, Milton Park, Abingdon, UK; 3 Cambea Consulting Ltd, 10 Beech Court, Hurst, Wokingham, UK; 4 Instituto Nacional de Investigación y Tecnología Agraria y Alimentaria (INIA), Madrid, Spain

## Abstract

The development and use of genetic technologies is regulated by countries according to their national laws and governance structures. Legal frameworks require comprehensive technical evidence to be submitted by an applicant on the biology of the organism, its safety to human, animal health and the environment in which it will be released. Some countries also require information on socio-economic and trade impacts. One of the key elements that assists decision-making under those legal frameworks is the use of risk assessments. The risk assessment paradigm of problem formulation based on risk hypothesis, and the assessment of plausible scientific pathways leading to potential environmental and human harms being realised, has been used widely to assess potential risks of genetic technologies to human health and the environment, from crops to mosquitoes. This paper uses the case study of a genetically modified self-limiting olive fly (*Bactrocera oleae*) for a first deliberate release in Spain to examine the regulatory processes and stakeholders involved in the assessment of risk. It is anticipated that existing risk assessment frameworks are equally applicable to gene drive technologies that may spread and persist in the environment and cross-national borders, but it is the governance structures surrounding the involvement of civil society in regulatory processes that must be administered in a more transparent and defined manner.

## Background

Insect pests present significant challenges to both agricultural systems and to public health, and promising solutions using genetic pest management strategies are now available or in advanced stages of development. Biological control using self-limiting genetics is one area in which commercial solutions are available and ready for deployment. The basis of self-limiting genetics is engineered lethality, whereby the genetically modified (GM) pests are expressly designed not to persist in the environment, and require sustained releases towards achieving control targets; they are effectively non-persistent in the environment by design.

Self-limiting *Aedes aegypti* developed by Oxitec Ltd. has advanced well past the validation phase and is available for commercial deployment where the regulatory environment is permissive. It has been demonstrated over the last 6–7 years that the release of mosquitoes that have been genetically modified to be self-limiting can provide substantial reductions in *Aedes aegypti* populations in target areas, with greater than 90% suppression of the *Aedes aegypti* population in the release area being achieved [1–3] without adverse consequences for human health or the environment.

Population replacement approaches and those employing gene-drive mechanisms are based on engineered environmental persistence. These approaches are entirely divergent in terms of the intended environmental fate of released insects, yet the existing risk assessment paradigm which has served to inform decision making under various global regulatory frameworks to date, is likely to serve well to characterize risk and inform risk management in all cases.

Self-limiting *Aedes aegypti* trials have been conducted in Cayman, Panama and Brazil and were approved using existing regulatory frameworks; frameworks that were largely designed for GM crops. In Brazil, Oxitec received approval to release on an unconstrained basis from National Technical Commission on Biosafety in 2014 [4]. Where existing regulatory frameworks for GM plants and other organisms are in place these have been applicable and adapted to the assessment of self-limiting insects. These regulatory frameworks are well characterized and already embedded at international level (e.g., The Cartagena Protocol on Biosafety). In these cases, the classical risk assessment paradigm and existing guidance frameworks for qualitative environmental risk assessment have proven sufficiently robust and comprehensive, and flexible enough for evaluating hazard and the potential for harm and subsequently informed risk management and regulatory decision making on what was a first for those regulatory systems.

Beyond the technical assessment used in the risk analysis, the element of stakeholder engagement, whereby risk communication is balanced with a presentation of the opportunities and benefits of new technologies must be strengthened in engaging with civil society in a transparent manner. This type of engagement has been previously called for in the EU context [5–8], but to date has not been addressed in legal frameworks, due to a lack of harmonization on the process and the risk of arbitrary decision making [9].

Communication of the potential benefits of new technologies, and not just the potential risk, should be more clearly embedded in the administrative processes, especially against existing alternatives (e.g chemical) pest control methodologies, which may have broader off-target effects. This would allow a more transparent, balanced and consistent approach to decision making. In the absence of such an approach governments are left with the messages which resonate the loudest; often those of special interest groups, many of which are fundamentally opposed to genetic technologies regardless of risk assessment conclusions or the benefits technology may bring.

The case study of an application for the self-limiting Olive Fly in Spain assessed under the requirements of European Commission (EC) Directive *2001/18/EC of the European Parliament and of the Council on the deliberate release into the environment of genetically modified organisms* herein illustrates that while the risk assessment at the core of science based decision making is robust, it is the surrounding governance framework including elements of stakeholder engagement and outreach which should be strengthened.

### Robust regulatory guidance for GM risk assessment

Regulations which cover the authorization and risk assessment for all activities carried out with GMOs in the European Union (EU) have been in force, and have seen implementation in member states since the early 1990s. Regulations establish notification procedures, governance structures and methodologies to perform a comprehensive risk assessment to characterize risks for human and animal health and for the environment. The interpretation of the EU regulatory framework for GMOs through guidance documents periodically published by the European Food Safety Authority (EFSA) collectively make the EU risk assessment framework for GMOs arguably the most rigorous and prescriptive regulatory system globally. Guidance is updated or issued in alignment with emerging issues and scientific and technical progress. Regarding genetically modified animals specifically, the European Food Safety Authority Guidance on the environmental risk assessment of genetically modified animals [10] provides detailed interpretation for the assessment of different animal taxa under the same generic framework. All these rules are based on the initial GMO definition set up in the EU Directives on contained use and deliberate release into the environment of GMOs. As defined under EU law, genetically modified organism (GMO) means:

‘an organism, with the exception of human beings, in which the genetic material has been altered in a way that does not occur naturally by mating and/or natural recombination’, using techniques of genetic modification such as recombinant nucleic acid techniques by plasmid or other vector system and their incorporation into a host organism; the direct introduction into an organism of heritable material prepared outside the organism including micro-injection, macro-injection and micro-encapsulation, and cell fusion (including protoplast fusion) or hybridization techniques where live cells with new combinations of heritable genetic material are formed through the fusion of two or more cells by means of methods that do not occur naturally’.

While different global frameworks may offer slightly different definitions, they all essentially derive from an interpretation aligned with the *Cartagena Protocol on Biosafety* definition of Living Modified Organism (LMO). While current applications in genetic pest management may fit the LMO definition, new genetic techniques may be utilized in the development of solutions to insect pest management in agriculture and public health, yet the risk assessment frameworks themselves remain evergreen and only supplemental guidance should be needed as new products come under regulatory scrutiny.

### Risk assessment framework

The classical risk assessment paradigm of problem formulation, qualitative risk assessment and the development of plausible scientific pathways and risk hypothesis leading to potential environmental and human harms being realized, has been used to date to assess potential risks of self-limiting GM insects to human health and the environment. It is anticipated that this robust methodology is equally suitable to gene drive or other genetic technologies engineered to persist in the environment or otherwise, although this has not yet been tested with submissions to regulatory agencies.

While the core principles of risk assessment are consistent across various global guidance documents*,* there is some variability in terminology, and in the presentation of processes. The classical environmental risk assessment (ERA) framework presented in the *Guidance Framework for Testing Genetically Modified Mosquitoes, World Health Organisation* [11] for example, follows a format exactly aligned with that presented in the *Guidance on the environmental risk assessment of genetically modified animals, European Food Safety Authority* [10]. The format is however somewhat different in the *Risk Analysis Framework, Office of the Gene Regulator, Australia* [12], and *Guidance on Risk Assessment of Living Modified Organisms and Monitoring in the Context of Risk Assessment* [13], yet the core principles are maintained.

**Fig. 1:**
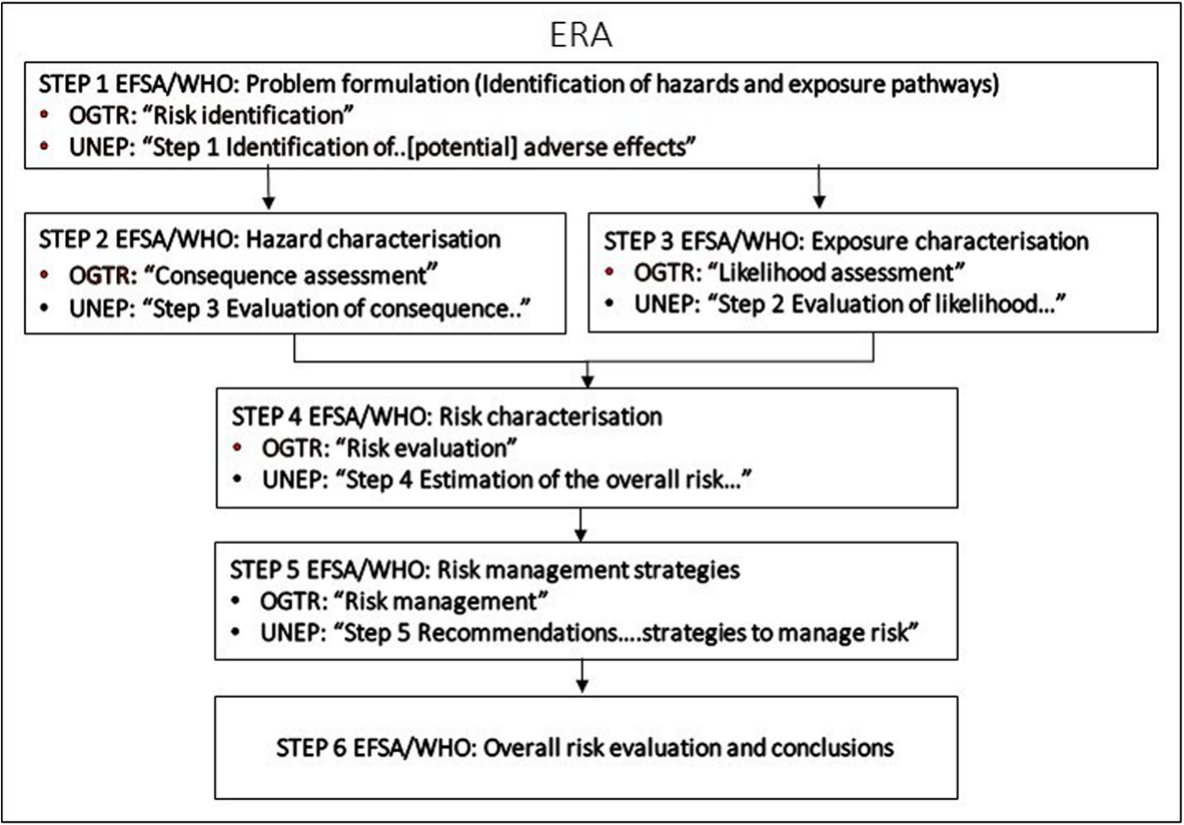
Key steps established in Guidance on the environmental risk assessment of genetically modified animals, European Food Safety Authority [10], Guidance Framework for Testing Genetically Modified Mosquitoes. World Health Organisation [11] alongside of the steps established in Risk Analysis Framework, 2013 Office of the Gene Regulator, Australia [12], and Guidance on Risk Assessment of Living Modified Organisms and Monitoring in the Context of Risk Assessment. UNEP/CBD/BS/COP-MOP/8/8/Add.1, September 2016 [13]

A structured and systematic approach following the six steps of ERA described in both EFSA 2013 and WHO 2014 enables a risk assessment following a single structured format, which systematically incorporates risk management. An alignment of various well-known ERA frameworks to demonstrate consistency across approaches in deriving risk conclusions is presented in Fig. 1. These approaches are aligned with the *Cartagena Protocol on Biosafety* as implemented in the European Union through Article 32 of the European Commission (EC) Directive *2001/18/EC of the European Parliament and of the Council on the deliberate release into the environment of genetically modified organisms.*

The term *risk analysis* is used to encompass all components of risk; namely, risk assessment, risk management and risk communication. OGTR (2013), WHO (2014) and EFSA (2013) guidance also include the element of *risk management* as part of the ERA process. The addition of the *risk management* element as part of a consolidated process in drawing overall risk conclusions serves as a useful extension to the core risk assessement as certain elements of risk management such as arthropod containment may be integrated as best practices in genetic pest control program design and thus may be given consideration in ERA process overall.

Risk assessment methodologies generally evaluate, as a first step, potential hazards and pathways to exposure (called the problem formulation step) so they may be identified and systematically evaluated against protection goals (i.e. what specifically in the environment must be protected). Protection goals may be derived from national policy initiatives for example, such as biodiversity protection, which may in turn have been derived from internationally binding agreements on environmental protection. ERA forms part of the overall risk analysis process in order to make informed decisions regarding the release and use of GM insects for pest management. The ERA is carried out using published data, study reports and other data generated through evaluations in contained use, and may include environmental releases that have been previously approved through regulatory frameworks. Additionally, scientific literature reviews and independent expert analysis may be considered in order to develop scientifically sound rationale in the overall assessment of risk.

Using the six steps of the ERA, seven areas of risk outlined in the EFSA 2013 specific guidance for GM insects are systematically evaluated. The approach of defining seven risk areas in EFSA 2013 provides a rigorous and systematic evaluation within a structured ERA framework that captures various examples and potential hazard scenarios.

The seven areas of risk identified in EFSA 2013 are:

1. Persistence and invasiveness of GM insects, including vertical gene transfer
2. Horizontal gene transfer
3. Pathogens, infections and diseases
4. Interactions of GM insects with target organisms
5. Interactions of GM insects with non-target organisms
6. Environmental impact of the specific techniques used for the management of GM insects
7. Impacts of GM insects on human and animal health

**Fig. 2:**
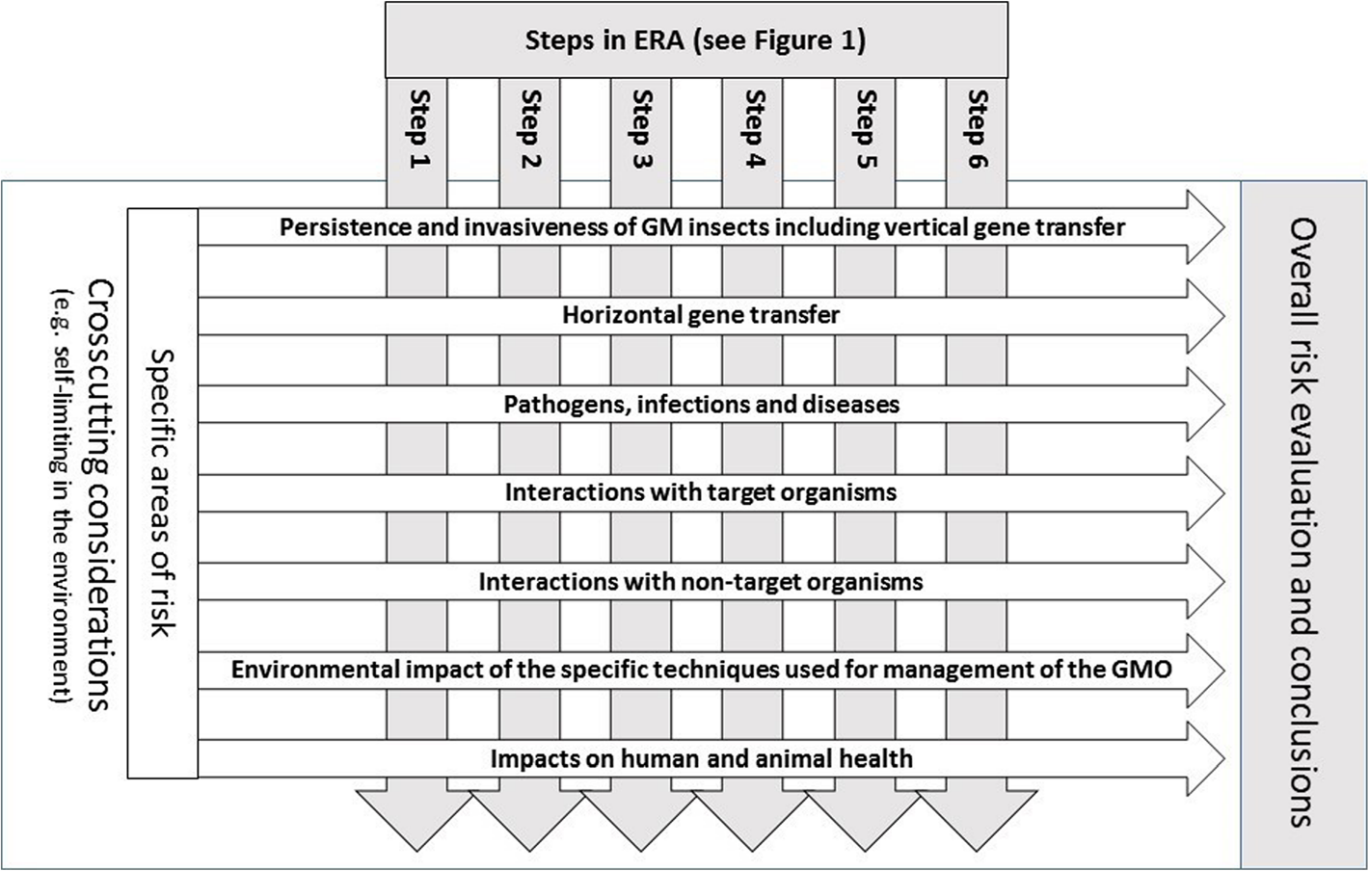
Structural representation of the ERA and inter-relation between the different elements. Steps in the ERA are taken from WHO 2014 and EFSA 2013, and specific areas of risk from EFSA 2013. Adapted from EFSA 2013

Figure 2 has been adapted from EFSA 2013 and represents how the EU ERA approach systematically ensures appropriate coverage of the seven areas of risk within a structured ERA framework.

In addition, generic crosscutting considerations may be taken into account throughout the ERA. In the case of self-limiting insect pests such as the Olive Fly discussed in the case study below, the principle crosscutting consideration is that it is self-limiting in the environment by design and the intended effect of the genetic modification results in a genetically modified pest that cannot establish in the environment, ultimately causing suppression of the local wild population in the receiving environment. WHO [11] provides a useful matrix which presents the self-limiting approach in the context of other potential applications of genetically modified insects broadly (Table 1).

**Table 1:** Reproduced from Guidance Framework for Testing Genetically Modified Mosquitoes. World Health Organisation [11] Table 1.1 Genetically Modified Mosquito (GMM) technologies currently under development. Note that the characterization does not describe all genetic systems in development, there are additionally “threshold-dependent” gene drive systems that have an element of local population control or reversal by design [14]

	Approach	
	Self- limiting	Self-sustaining
Population suppression	Modification reduces the number of progeny	Modification reduces the number of progeny
	Possesses either no gene drive or weak drive that will pass the modification through only a limited number of generations	Possesses strong gene drive
	Not intended to persist in the absence of continued releases	Intended to spread the modification indefinitely or until the mosquito population is eliminated
Population replacement	Modification limits pathogen replication, thereby reducing transmission	Modification limits pathogen replication, thereby reducing transmission
	Possesses weak gene drive that will pass the modification through only a limited number of generations	Possesses strong gene drive
	Intended to spread the modification through the population indefinitely	Intended to persist only until diluted out of the population

Additionally, EFSA 2013 considers potential applications for insect vector control, agricultural pest management as well as the enhancement of production systems (e.g. honey bees, silk worms). Within the category of insect vector control, EFSA 2013 further identifies potential applications using GM insects for both population suppression or population replacement as in the WHO 2014 reference (Table 1).

The Oxitec GM Olive Fly is designed to be self-limiting in the environment and is non-persistent by design. GM pest insects for population suppression must be considered in the context of the intended non-persistent design, whereas strong gene drives intended to self-sustain, persist, and drive through the target population must be given appropriate consideration early in the problem formulation of the ERA. Similarly, regardless of the methodology used to produce an insect biological control agent, whether it be GM or otherwise, the classical risk paradigm, with the areas of risk established in existing guidance such as EFSA, is sufficiently rigorous to identify hazards, and the potential for harms to the protection goals.

### Problem formulation

Problem formulation is a systematic and transparent methodology for identifying potential hazards, and potential exposure pathways which could ultimately lead to harm being caused to the entities that require protection. The problem formulation provides a direction for the ERA and indicates where further data may be required. It is used in a variety of risk assessment scenarios such as for chemicals and genetically modified crops, and should be equally applicable to any novel living organism whether narrowly defined as a GMO, or developed using new and emerging genetic techniques.

A hazard may simply be defined as: the potential of an organism to cause harm to human health and/or the environment. This is consistent with various sources of international guidance and taken from Guidance on Risk Assessment of Living Modified Organisms and Monitoring in the Context of Risk Assessment, UNEP/CBD/BS/COP-MOP/8/8/Add.1, September 2016 [13]. The EFSA glossary- taxonomy terms 1 defines hazard as a substance or activity which has the potential to cause adverse effects to living organisms or environments. The EFSA Scientific Opinion on Risk Assessment Terminology [10], in comparing definitions from international standard setting organisations (CAC, OIE, IPPC) identified the common element in defining a hazard as something that has a potential to cause an adverse health effect or to be injurious to target populations. A hazard thus considers the characteristics of the potential adverse effect, independent of the likelihood of exposure. Whereby harm in the context of an ERA may be defined as an adverse outcome or impact [12] and accounts for the exposure pathway.

A problem formulation carried out as Step 1 in each of the seven specific areas of risk in Fig. 2 will serve to identify the potential for harm, regardless of the persistence profile of the insect (e.g. self-limiting or gene-drive), or whether the product of a technology fits the GMO/LMO regulatory trigger. In order to evaluate the magnitude of potential harm, they must be linked to assessment endpoints, derived from protection goals in the receiving environment. Assessment endpoints allow the formulation of a risk hypothesis, which can be tested through a systematic, methodological approach.

Protection goals are generally aligned with principles common to risk assessment guidance frameworks which generally serve to assess whether the GMO may adversely affect human health, animal health or the environment. Examples of generic protection goals:

1. Protection of human and animal health - ensuring that humans or non-target animals are not harmed by the release.
2. Protection of biodiversity and ecosystem services - ensuring that irreversible harm or harm that cannot be mitigated does not occur due to the release
  - Populations of charismatic or protected species which are likely to inhabit or have overlapping habitat with the areas where releases will take place.
  - Populations of key species that are the sole providers of, or key contributors to, ecosystem services in the areas where releases will take place.

**Fig. 3:**
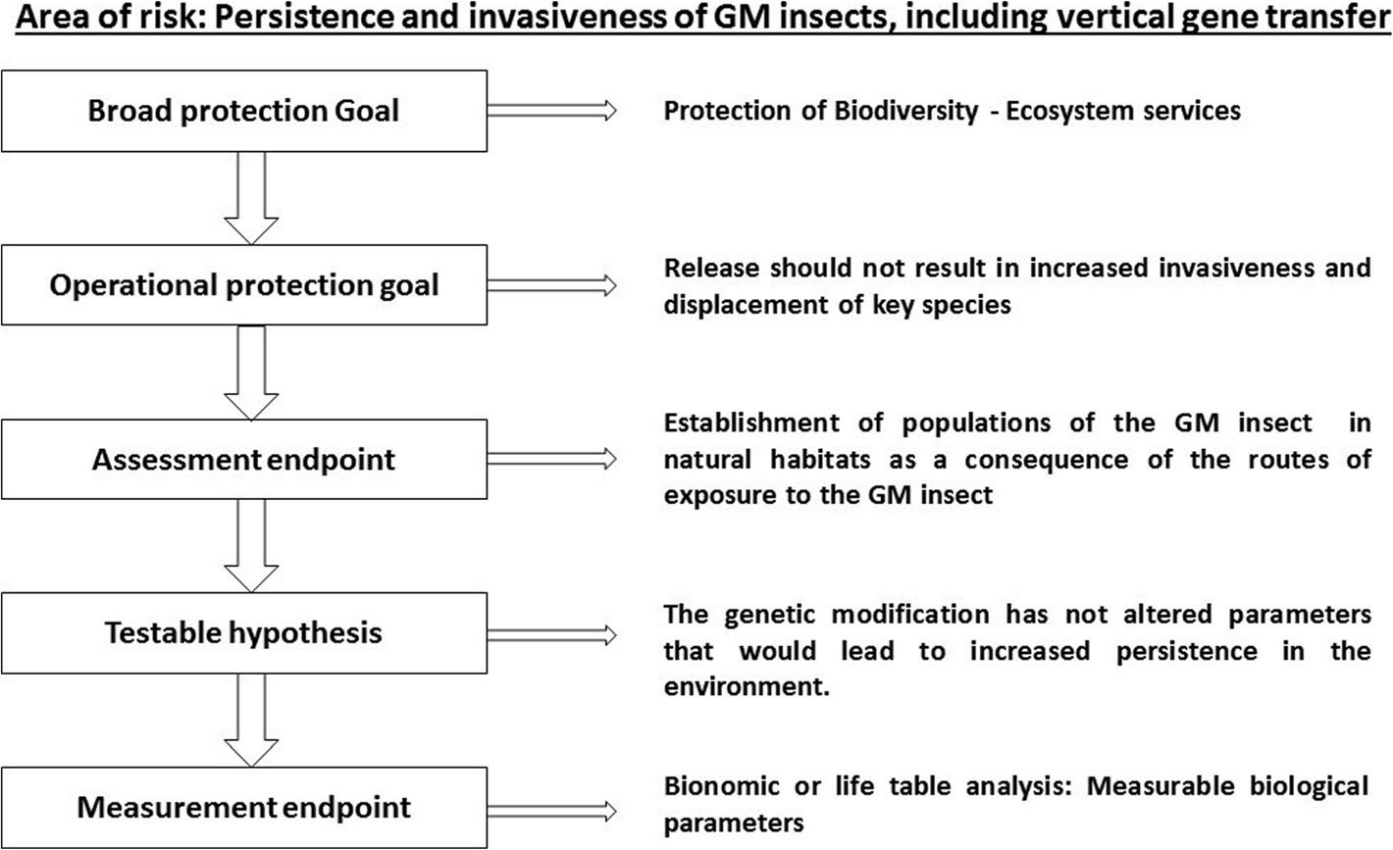
Process of deriving measurement endpoints from broad protection goals in risk assessment. Theoretical example provided for a generic GM insect in the defined risk area of persistence and invasiveness

Broad protection goals are examined individually in the context of each of the specific areas of risk and ultimately refined into assessment endpoints. A risk hypothesis may then be formulated, and tested towards defined measurement endpoints, which are in some cases quantifiable, and which can act as indicators of change, and thus measures of hazard and exposure. Fig. 3, adopted from EFSA (2013) illustrates this principle with a theoretical example for a generic GM insect examined under the persistence and invasiveness area of risk. The model should be applicable broadly regardless of the technology used to introduce the novel trait(s).

Through the problem formulation (Step 1), potential hazards and pathways to exposure may be identified and systematically evaluated against protection goals. The process of developing a risk hypothesis, and assessment and measurement endpoints, may reveal that there is adequate information to formulate a conclusion that there is no plausible pathway to harm, and that further hazard and exposure characterization (Steps 2 and 3) is not required, or conversely may identify the need for further characterization. The following questions are considered in identifying where further characterization is needed (adapted from [12]):

- Is the hazard or exposure pathway attributable to the genetic modification?
- Is there a plausible and observable pathway linking the GMO to the potential hazard or pathway to harm?
- Is the potential hazard or pathway to harm substantive? (i.e. can the magnitude be estimated in the problem formulation step?)

If specific hazards are identified in the problem formulation step and deemed as requiring further characterization they can be subject to an initial qualitative evaluation, and in certain cases where feasible, a quantitative evaluation may be warranted. A quantitative approach is possible mostly where parameters can be readily measured and compared to baseline data from an appropriate comparator; this is most often the case where there is a history of accumulated data. A qualitative approach is however applied frequently when dealing with interactions between biological systems due to their complex and dynamic nature. Measurement endpoints for example may often be evaluated through a qualitative overview of current opinion rather than specific experimental measurements.

The structure of the problem formulation considering elements of the guidelines proposed by Wolt and colleagues [15], at its core should serve broadly to inform risk analysis for products envisioned for genetic pest management strategies whether derived through GM technologies, or those technologies falling outside of this narrow definition. The case study of the Oxitec self-limiting GM Olive Fly below outlines how the EU framework for environmental risk assessment was adequately robust to assess risk, but a more structured approach to the governance of stakeholder and public engagement is needed as a complement in bringing new technologies to use for genetic pest management.

### Public engagement

In the spirit of fostering open communication and transparency, the legally binding obligation for public consultation processes to be undertaken to inform the public of deliberate releases or placing on the market of GMOs in the EU is clearly established in Directive 2001/18/EC of the European Parliament and of the Council. There is a clear obligation to carry out a consultation for public comments through publishing summary information of any proposed release for a minimum of 30 days. Member state national websites provide a tool for the dissemination of this information to the public within the individual member state, and the established process to inform member states in the EU community more broadly is through the website of the European Commission Joint Research Centre (JRC). The Summary Notification Information Format (SNIF) establishes a standardized format for member states to ensure consistency through the submission and posting of required information for public comment.

The express purpose of the JRC SNIF notification web pages, (managed by the JRC on behalf of the Directorate General for Health and Food Safety), is to publish information and to receive comments from the public regarding notifications submitted by the applicants to the Member States Competent Authorities about deliberate release field trials and placing on the market of genetically modified organisms, as defined in Directive 2001/18/EC. Following the 30-day consultation period, the Commission sends the comments received directly to the Member state competent authority for analysis. Where comments concern applications for GM food and feed under Regulation (EC) 1829/2003, scientific comments are sent to the European Food Safety Authority (EFSA) who checks their impact on the previously prepared EFSA scientific opinion. The final assessment report is also subject to a 30-day public comment period.

Public consultation in this context is by design intended to elicit commentary of a scientific nature in which only new information about risk assessment which warrants further consideration in the overall risk analysis is accounted for in the formal process. The process is intended to ensure decision making on GMOs is robust and science-based and appears to achieve this objective with transparency. Comments from the general public which fall outside of the scope of evidence based decision making however, such as issues around public acceptance generally and ethical considerations do not have a transparent mechanism for incorporation into the decision-making process. It is thus not clear in this context what the mandatory public consultation process serves to achieve beyond the narrowly-defined scope of ensuring a comprehensive review of the science based evidence at the time of publication. The general public thus may be under the illusion that the process is intended to be much more encompassing that it is in practice, a topic which has been extensively reviewed [16].

### Case study with GM olive flies under the EU regime, via Spain as the lead member state

In the specific case of Spain, the EU Directives on the contained use and deliberate release into the environment of GMOs have been transposed through a National Law [17] and a Royal Decree [18] which established the legal regime governing the contained use, deliberate release and marketing of GMOs. The Spanish Act also created the Competent National Bodies for authorizing (Inter-ministerial Council of GMOs), assessing (National Commission on Biosafety) and communicating to the Civil Society (Participation Committee), all activities carried out with GMOs and set out the distribution of competencies among Central Government and the Spanish Regions. The Spanish regions are, in most of the cases, the Competent Authorities for approving experimental field trials for deliberate release of GMOs in their territories. The risk assessment framework in Spain thus follows that presented in Fig. 1 as established in European Commission (EC) Directive 2001/18/EC of the European Parliament and of the Council on the deliberate release into the environment of genetically modified organisms.

In Spain, the communication body, referred to as the Participation Committee above, is responsible for providing the results of the risk assessment for deliberate release of GMOs in the environment to the Stakeholders, when the Central Government is responsible for authorizing it. In this committee, doubts or concerns can be expressed to the Competent Authority before a final decision is taken on the trial. The distribution of competencies responsible for sharing environmental issues in Spain is such that the regional authorities are responsible for the authorization and management of the deliberate releases carried out with GMOs in their territories, including communication procedures. In this sense, each Spanish region manages in its own way the process of public information and the involvement of its community stakeholders. For this reason co-ordination of communication efforts can be challenging.

In December 2012 Oxitec Ltd. applied to Catalonian regulatory authorities for permission to carry out a netted field evaluation of a GM self-limiting Olive Fly strain (OX3097D-Bol). The Olive Fly (*Bactrocera oleae*) is the single most important economic pest for olives, causing widespread crop damage and significant financial losses to Europe’s olive farmers. There are approximately 5 million hectares of olive tree farmland in the European Union and more than half of the world’s supply of olive oil is produced in Spain. The Olive Fly has become increasingly resistant to pesticides, while at the same time many efficacious chemical controls have been or are being phased out in the EU. The OX3097D-Bol self-limiting strain of Olive Fly was proposed as a novel approach to controlling this damaging agricultural pest. The field testing protocol proposed would have been the first outdoor trial of a genetically modified insect in the EU as a follow-up to earlier indoor caged trials in which OX3097D-Bol was able to eliminate wild-type Olive flies in less than two months [19].

The primary objectives of the proposed netted field trial were to:

1. establish the performance of OX3097D-Bol males when competing with wild males for wild females;
2. gather information on the longevity of OX3097D-Bol in a field environment, and
3. evaluate different release methods for adapting release rates to wild prevalence.

The netted field trial was proposed for a period of 8 weeks (releasing OX3097D-Bol once or twice a week) within the timeframe of April 2013 to April 2014. The trial site was in Tarragona, on a research station, in managed agricultural land, free of grazing livestock approximately 8 km from the port at Tarragona and 157 km from Aigüestortes i Estany de Sant Maurici National Park. The main agricultural crops in the area are olive and hazelnut trees, but there are also other crops (almonds, walnuts, carobs, pistachios, figs, apples, pears, peaches and vineyards).

### The Oxitec genetically modified olive fly- OX3097D-Bol

The genetic basis of the self-limiting trait and the fluorescent marker gene in OX3097D-Bol has been previously described [19]. Briefly, two traits have been introduced into OX3097D-Bol on a single inserted DNA construct:

1. a conditional female specific self-limiting trait through the expression of the tTAV protein (a synthetic fusion protein of sequences of viral and bacterial origin). tTAV thus acts as a tetracycline regulated switch which confers conditional cell death in females in the absence of tetracycline or its analogues, yet enables the mass rearing of the Olive Fly in the laboratory through the inclusion of tetracycline in the rearing medium.
2. a fluorescent marker for use in field monitoring through expression of the DsRed2 protein (from a coral, Discosoma Sp.) (Clontech). The marker gene enables the detection of OX3097D-Bol in the field and allows the evaluation of the dissemination of OX3097D-Bol genes resulting from the release of males.

Released male OX3097D-Bol are thus not biologically sterile as they will produce offspring, however as the female offspring do not live past the larval stage, the self-limiting effect results in a reduction of the population with each generation. Ultimately population control is realised through a reduction of the wild female population.

### Initial proposed study protocol

The total netted release site proposed was within a small plot of olive trees of different varieties, and split into 6 small treatment sites (6 small cage areas, 144 m^2^ each, with 4 olive trees inside each), all totaling an area less than 1000 m^2^. The releases of OX3097D-Bol would only be within the netted areas with initial proportion of releases 1:1 for GM Olive Fly and wild populations.

Control and monitoring measures of the release were proposed. Pre-release monitoring would commence after approval was given and in advance of the trial. Monitoring would continue throughout the trial in addition to a proposed 4-week period of post-release monitoring.

Specific methods planned for monitoring OX3097D-Bol during the trial were as follows:

1. standard Olive Fly traps would be set up at intervals outside of the netted release sites to monitor for the presence of any escaped OX3097D-Bol in the vicinity of the netted cages;
2. standard Olive Fly traps would be set up within the netted cages and would be checked periodically to monitor the population of the olive flies within;
3. fluorescent scoring of samples of olive flies from the traps would detect the presence of the OX3097D-Bol genetic construct;
4. immature stages may be monitored by sampling infested olive fruit;
5. the vertical transfer of genetic material can be detected in the Olive Fly by screening for fluorescence as the exchange of gametes is limited to Olive Fly by mating behaviours, and;
6. presence of the genetic material may also be verified by molecular methods.

If OX3097D-Bol were detected at the release site at the end of the monitoring period then an approved insecticide was proposed for treatment. In any case, the applicant indicated that the OX3097D-Bol males are not expected to persist in the environment, and they would not live beyond their own short lifespan.

Regarding waste management, any eclosion and release materials used at the eclosion facility would be frozen at − 15 °C or below for 12 h prior to disposal through the usual waste channels. Emergency measures were proposed in the event of an inadvertent escape during transportation although OX3097D-Bol are not capable of establishment in the environment due to the presence of the self-limiting trait. Any large-scale accidental release could be treated with an approved insecticide. Any unexpected persistence of the Olive Fly would be detected by site monitoring and, if necessary, approved insecticides be applied.

### Environmental risk assessment (ERA) provided by the applicant

An ERA compliant with EU and Spanish law was provided by the applicant, along with a Summary Notification Information Format (SNIF) which was made public via the established mechanism of the European Commission and the Spanish government. The main conclusions of the ERA were:

- Genetic stability: OX3097D-Bol was developed over 3 years (approx. 45 generations). No signs of instability within the genetic trait were observed (morphological evaluation, PCR analysis with the known flanking sequences and assessment of the female lethality trait).
- Persistence and invasiveness: The mode and rate of reproduction has not been altered, however the intended effect of the modification is to confer a selective disadvantage on the females (i.e. death) in subsequent generations.
- Gene transfer from the OX3097D-Bol males to the wild females is likely to result in the death of the female progeny in the release site as the conditional lethality trait female-specific. Males from such crosses are not anticipated to have an altered lifespan in comparison to the unmodified Olive Fly.
- Toxicity/allergencity: The tTAV protein which confers the conditional female specific self-limiting trait has been compared to known toxic and allergenic sequences and was found not to encode any sequence homologous to a toxin or allergen. The fluorescent marker DsRed2 has no known toxicity or allergenicity.
- Interactions with target organisms: All female offspring resulting from crosses will not survive to adulthood as there is a lack of the tetracycline supplement required to suppress the self-limiting trait in the release environment.
- Interactions with non-target organisms: no significant interactions are anticipated. If the OX3097D-Bol is eaten by predators present at the release site the inserted genetic traits are not anticipated to have any toxic effect.
- Impact on biogeochemical processes: The two novel expressed proteins are likely to breakdown into constituent amino acids at the release site. An influx of dead adult males and any change in the overall Olive Fly population is not considered to have a lasting detrimental impact on the decomposer population.
- Human and animal health: There will be a negligible risk associated with the release of the OX3097D-Bol Olive Fly.

The overall conclusion for the ERA by Oxitec was: ‘No significant interactions are anticipated. The modification is limited to the Olive Fly by reproductive barriers’.

### Ensuing technical dialogue

In May 2013, the Catalonian Competent Authority asked for the ERA report to be evaluated by the Spanish National Commission on Biosafety (CNB). During the assessment process, the CNB requested the applicant supply additional information regarding biosecurity controls and containment for the trial, as well as additional scientific evidence to support the conclusions of the ERA. The CNB’s main concerns related to: site biosecurity and containment; the effectiveness of the self-limiting trait; the potential for horizontal gene transfer, and; the unexpected adverse effects on non-target organisms.

After the preliminary ERA by the CNB in June 2013, additional information was requested of the applicant including:

1. Regarding site-biosecurity:

- the exact geographical location of the caged installations, detailed characteristics of the netting, its construction and installation, how deep it was buried into the soil at the perimeter;
- Enhancement of the containment measures compared with the initial proposal (i.e. clothing would be sterilized or incinerated after using).

2) Regarding additional data and evidence to support risk assessment conclusions:

- More scientific data regarding the possibility of horizontal transfer of genetic material (HGT) due to the mobility of the transposable element (TE) included in the genetic trait;
- Additional evidence to support the penetrance of the self-limiting trait (i.e., that the hemizygous female offspring containing the gene construct passed from male OX3097D-Bol do not survive as intended);
- Additional data on the safety of the t-TAV protein for non-target organisms and;
- Further information about the levels of tetracycline into the environment.

In October 2013, a meeting between the Competent Authority, members of the CNB and Oxitec was held in Madrid to clarify further questions raised at the CNB meeting. As a result of this meeting, in November 2013, Oxitec sent a letter to the CNB informing of the withdrawal of the notification to allow time to finalize the additional information requested by the CNB. The public posting of the SNIF was withdrawn and thus no public comments on the application were evaluated. The SNIF posting, while it was active, had however generated attention from various special interest groups, principally from those opposed to genetic technologies, and the ensuing result was increased media focus.

In May 2015, the Applicant again submitted a Notification to the Catalonian CA with the purpose of performing the trials during the period from July 2015 to July 2016. In the new application, amended site biosecurity provisions as well as the studies and additional scientific information required by the CNB were provided.

1. Regarding site-biosecurity:

- the net perimeter was to be buried 0.5 m into the ground to avoid the entrance of insects or small mammals.
- Netting specifications were provided with a mesh size of 0.75 mm × 0.39 mm, preventing the movement of insects of greater size
- The entrance (only authorized personnel) would be through a vestibule with double doors where personnel change its clothes.
- Traps would be deployed before, during and post-release both inside and outside the netting.

2) Regarding additional data and evidence to support risk assessment conclusions:

- Additional scientific literature was provided on the lack of mobility of *piggyBac* TE concluding that it posed a negligible or very low risk.
- A study with larger numbers of insects was provided by the applicant to improve the statistical power of the penetrance of the self-limiting trait.
- It was concluded that the level of tetracycline was difficult to assess in the environment as it is very sensitive to light and temperature, thus the likelihood of environmental exposure was considered to be negligible.
- Three additional non-target studies on specific Olive Fly terrestrial predators and parasitoids were presented where no adverse effects were noted.

As in the previous application, subsequent to the official submission, the relevant information supplied in a SNIF was made public via the established mechanism of the European Commission and the Spanish government. Upon receiving additional data and evidence, and further information on site biosecurity, the CNB was satisfied although there were outstanding elements yet to be addressed relating to physical and procedural biosecurity measures. It was considered additional conditions would be required including: (i.e. double net to prevent risks in case of uncontrolled incidents (hail, animal bites, etc.); management in the case of incident during transportation; potential use of a vacuum cleaner in the vestibule and heat treatment of bags and; one year post-release monitoring, removal and decontamination of olives, among others.

Subsequently further dialogue was pursued between Oxitec, and the Catalonian CA whereby additional biosecurity measures such as ground netting were proposed and physical and procedural containment measures for the transport of OX3097D-Bol were clarified. Despite demonstrating containment principles for the proposed outdoor trial consistent with those employed in various global regimes for plant pest containment, the feasibility of anything other than a trial under the EU contained use regulations was unlikely. On 5 August 2015, after some consultation between Oxitec and the Competent Authority, Oxitec formally withdrew the application. The Catalonian CA asked the CNB to close the assessment procedure, and the SNIF notification was removed from the public domain. No public comments were evaluated. As in the case of the previous application, negative attention from special interest groups, as well as a range of perspectives presented by the media was prevalent in the public domain. In October 2015, the Catalonian CA sent Oxitec a formal notice of the application’s withdrawal.

### Stakeholder outreach

In the context of the risk analysis more broadly, other ethical, political and socioeconomic issues had been considered, however while contact was pursued with various stakeholder groups, no formalized mechanism to evaluate outputs was followed. Extension workers and staff from the research station where the trial was to be conducted were briefed and prepared in speaking with local producers and stakeholders, but no overarching coordinated strategy was implemented in collaboration with the Spanish national and Catalonian regional governments.

Non-governmental organisations (NGOs) in Spain, especially environmental organisations, were strongly opposed to the release of GM insects into the environment under research and marketing purposes, based principally on organisational ideology. A broad discussion was generated in the media regarding the proposed trial. The CNB consulted with the Spanish Authorities on Plant Health and the olive oil sector regarding their opinion on the technology and perceived potential market and agronomic impacts of an OX3097D-Bol trial. Spanish Authorities on Plant Health and farmers associations generally considered the use of OX3097D-Bol an interesting alternative strategy for biological pest control (except for organic farmers who held principles embedded in organic regulations). In the case of the olive oil sector, farmers showed deep concern about the possible market impact of introducing any genetically modified component into production streams, due to the lack of regulatory predictability in this emerging area of biological control. There was a perception that EU labelling requirements could be triggered due to potential remnants of GM material inside the olives, and that it could be detrimental to marketing efforts.

Meanwhile the Catalonian CA remained concerned about the media’s reaction and public opinion, the perceived potential impact on the environment and unpredictable commercial consequences. At this stage, although the CNB has consulted with some sectors involved, a public consultation was not finally carried out by the Catalonian Competent Authorities.

## Conclusions

Robust risk assessment frameworks are well established for GMOs, and the EU has arguably one of the most rigorous and prescriptive frameworks based in both legislation and associated guidance documents. Established principles of risk assessment should serve to effectively identify and characterize hazards to inform risk management and risk communication for genetic pest management approaches with widely variable environmental persistence profiles such as self-limiting insects, and those with gene drive mechanisms. As these new technologies are presented to risk assessors under various regimes globally, the lack of precedent and guidance in applying existing risk assessment frameworks for the new product classes will challenge decision making. Global collaborative networks of researchers, product developers and risk assessors must be strengthened and leveraged across relevant disciplines to reduce uncertainty to the extent possible in formulating risk conclusions on new technologies.

At the European level a structured and balanced stakeholder engagement process is needed on the use of emerging genetic technologies, both in the context of risk communication and their benefits for society. The processes for accounting for input from civil society are not defined in a transparent manner. The proposed netted trial of a GM self-limiting Olive fly in an agricultural research setting in Spain was an early stage developmental trial intended to generate data to help evaluate its future potential and provide evidence to further validate the biosafety profile. This was the first time a submission of a self-limiting GM pest control technology was evaluated in the EU. While stakeholder engagement activities were undertaken in the broader risk analysis, considering the capacity of established NGOs to influence public opinion on genetic technologies, a more transparent procedure should have been followed in Spain more effectively using all communication channels available to increase communication and debate between all stakeholders involved to both regional and national levels. The engagement of all interested parties involved in decision-making is highly desirable, yet the consideration given to the outcomes of these processes needs to be well defined such that engagement efforts receive appropriate attention, resources, and advanced planning.

Additionally, the regulatory oversight of the potential for potential residual GM material in food products in this context is not clearly established in EU regulation, as a GM insect used in a genetic pest management system itself is not regulated as a food product in the context of the labelling regulations. Regulatory policy in this area at the EU level needs to be further explored such that market reaction is predictable to better inform overall risk analysis. As this element was not adequately scoped out in response to it being raised through engagement activities, stakeholders are left with presumptions that are not founded in fact.

The interplay between various stakeholders is generally complex for genetically modified organisms, and perceptions about both environmental and commercial risk may be disproportionate and uninformed by robust analysis. While current qualitative environmental risk assessment frameworks are sufficiently robust for existing and emerging technologies, we recommend that a highly participatory and proactive governance framework needs to be strengthened to consider how ethical, political and socioeconomic issues surrounding new technologies inform decision making in a balanced and proportionate manner.
